# Commentary: Metacognition and Perspective-Taking in Alzheimer's Disease: A Mini-Review

**DOI:** 10.3389/fpsyg.2018.02010

**Published:** 2018-10-22

**Authors:** Rosalba Morese, Mario Stanziano, Sara Palermo

**Affiliations:** ^1^Faculty of Biomedical Sciences, Institute of Public Health, Università della Svizzera Italiana, Lugano, Switzerland; ^2^Faculty of Communication, Culture and Society, Università della Svizzera Italiana, Lugano, Switzerland; ^3^Neuroradiology Unit, Azienda Ospedaliera Universitaria “Città della Salute e della Scienza di Torino”, Turin, Italy; ^4^Department of Psychology, University of Turin, Turin, Italy; ^5^European Innovation Partnership on Active and Healthy Ageing (EIP-AHA), Brussels, Belgium

**Keywords:** metacognition, self-awareness, cognitive awareness model, anterior cingulate (ACC), Alzheimer's disease

The neurocognitive approach points out how reduced self-awareness is related to cerebral disorders, particularly concerning focal lesions, emotional and motivational determinants, and concurrent neuropsychological impairments (Palermo, 2017). Given its consequences on patients' quality of life, caregivers' burn-out, compliance with treatment, and prognosis, reduced self-awareness has received increasing attention on the part of neuroscientists (Fernández-Calvo et al., 2015). Indeed, a reduction in self-awareness has been found to be associated with a decline in help-seeking behavior and compliance with medical treatment, presumably because of a reduction in motivation (Palermo, 2017).

In their brilliant mini-review, Bertrand and colleagues drew attention to the fact that neurological and neuropsychiatric patients can present severe—and often underestimated—impairments in metacognitive abilities that may result in “*a lack of self-awareness of deficits*.” Indeed, executive-metacognitive dysfunctions have been formerly associated with loss of self-awareness across major neurocognitive disorders likely due to Alzheimer's Disease (AD) and frontotemporal dementia (Amanzio et al., 2011, 2013, 2016), Acquired Brain Injury (Palermo et al., 2014), Bipolar Disorder (Palermo et al., 2015) and Parkinson's Disease (Amanzio et al., 2014; Palermo et al., 2017a,b).

Focusing on AD, Bertrand et al. (2016) dwelt on the association between *metacognition* and *perspective-taking* as a key aspect for improving quality of life in AD patients. These authors specified that “*metacognition refers to the monitoring and regulation of cognitive processes and its impairment can lead to a lack of self-awareness of deficits, or anosognosia*” (Bertrand et al., 2016, p. 1).

Anosognosia and reduced self-awareness are frequently used as synonyms in the literature and an overlap between them has been highlighted. This is a delicate question because according to the interpretation given to those terms, the methods of detection and interpretation of the phenomenon could change. This topic was recently treated by Mograbi and Morris (2018). In their interesting Discussion Forum, the authors not only emphasize how the term anosognosia continues to be the most used in the neurological field, but propose a glossary of labels that enhances the differences in use and interpretation of the epiphenomena to which they refer.

In our opinion, the notion of “self-awareness” is not entirely concerned with the concept of “anosognosia,” since the former is a complex neuropsychological notion, defined as “*the ability to consciously process information about ourselves in a manner that reflects a relatively objective view while maintaining our unique phenomenological or subjective sense of self* ” (Prigatano, 1997, p. 301). Indeed, awareness of illness is above all a form of self-knowledge and a higher-order cognitive function covering information about the state of the disease, its functional consequences, the way in which it affects the patient and influences his/her interaction with the environment (Palermo, 2017). In support of this theoretical framework, Stuss et al. (2001) and Stuss and Anderson (2004) argued that there are several types of self-awareness (the so-called “consciousness” in their theoretical framework), organized hierarchically, which should be differentiated.As we have previously outlined, they proposed different elements in a four-level hierarchical framework of awareness, “*where the processing in turn operates in a modular manner at any level of the hierarchy, so that damage in one functional domain may result in a different kind of reduced self-awareness*” (Palermo et al., 2014, p. 541). These four types of self-awareness have their own neural underpinnings in distinct substrates, with the lower levels related to a selective disturbance of knowledge [anosognosia] and the higher levels related to self-awareness, perspective-taking and Theory of Mind, with an emphasis on the role of the frontal and medial prefrontal brain areas (Palermo, 2017). Indeed, the cognitive processes involved in metacognitive-executive functions and Theory of Mind have been associated with complex ecological contexts that require the recruitment of the anterior cingulate cortex and the ventromedial prefrontal cortex (Morese et al., 2016). Moreover, (O‘Keeffe et al., 2007). proposed that any disturbance in monitoring, cognitive flexibility and response inhibition has the potential to distort patients' foresight (and thus self-awareness).

Bertrand et al. (2016) suggested that the Default Mode Network may be useful to shed light on the role of perspective-taking in awareness of deficits in AD. They presented this relationship by referring to the Cognitive Awareness Model (CAM), which incorporates a comparator system within the central executive to detect mismatches between a personal database and experience of failures and successes (Agnew and Morris, 1998). When a discrepancy is found, a signal is sent out to the metacognitive awareness system (the fourth level in the model by Stuss et al., 2001), enabling a conscious experience of failure/success. If the executive system does not work properly, the comparator mechanism may not detect mismatches, and subsequently experienced failures may not produce any metacognitive output or conscious awareness, leading to an “executive unawareness” in the CAM (Agnew and Morris, 1998).

This model was applied by Amanzio et al. (2011), who addressed the relationship between executive dysfunctions and reduced self-awareness in AD using fMRI and an event-related specific executive task (a Go/NoGo task adapted from Braver et al., 2001; Palermo et al., 2018). Their findings suggest that lower self-awareness in AD patients is linked to reduced functional recruitment in the medial prefrontal circuit, in particular in the Anterior Cingulate Cortex (ACC) (Amanzio et al., 2011). Furthermore, response-inhibition, self-monitoring, and set-shifting dysfunctions have to be considered prominent features of reduced self-awareness in AD patients (Amanzio et al., 2011, 2013), while apathy and disinhibition would seem to be the first relevant behavior changes in AD patients with reduced self-awareness (Amanzio et al., 2011).

In conclusion, reduced self-awareness is often observed in AD patients who have suffered major damage to the central executive system. This could be due to the fact that the central executive system is a metacognitive facility of interest in controlling the flow of information in tasks requiring initiation, planning, set-shifting, strategy allocation, monitoring and inhibition (Amanzio et al., 2011, 2013). As indicated by Bertrand et al. (2016), the CAM is one of the most fruitful models for understanding the *irrefutable liaison* between executive system dysfuntion, metacognitive disabilities and the occurrence of reduced self-awareness. The issues raised in this commentary have implications in considering executive-metacognitive disabilities as a neuropsychological marker for reduced self-awareness in AD.

We would like to conclude by drawing attention to the fact that this phenomenon is worthy of clinical investigation. Indeed, distinct major neurocognitive disorders, brain pathologies and mood disturbances exhibit executive-metacognitive abnormalities as part of the anomalies of overlapping neural pathways. In this context, ACC hypofunctionality seems to represent one of the relevant underpinnings of a lack of self-awareness (Amanzio et al., 2011; Palermo et al., 2014, 2015).

## Author contributions

The commentary is based on a concept developed by SP who performed the review and critique processes as PI. All the authors listed have contributed to writing the commentary. All authors have approved the submission of the manuscript.

### Conflict of interest statement

The authors declare that the research was conducted in the absence of any commercial or financial relationships that could be construed as a potential conflict of interest.
