# Corrigendum: Commentary: Metacognition and Perspective-Taking in Alzheimer's Disease: A Mini-Review

**DOI:** 10.3389/fpsyg.2020.02157

**Published:** 2020-10-20

**Authors:** Rosalba Morese, Mario Stanziano, Sara Palermo

**Affiliations:** ^1^Faculty of Biomedical Sciences, Institute of Public Health, Università della Svizzera Italiana, Lugano, Switzerland; ^2^Faculty of Communication, Culture and Society, Università della Svizzera Italiana, Lugano, Switzerland; ^3^Neuroradiology Unit, Azienda Ospedaliera Universitaria “Città della Salute e della Scienza di Torino”, Turin, Italy; ^4^Department of Psychology, University of Turin, Turin, Italy; ^5^European Innovation Partnership on Active and Healthy Ageing (EIP-AHA), Brussels, Belgium

**Keywords:** metacognition, self-awareness, cognitive awareness model, anterior cingulate (ACC), Alzheimer's disease

An author name was incorrectly spelled as Stanziano Mario. The correct spelling is Mario Stanziano. Also, there was an error regarding the affiliations for authors Rosalba Morese and Sara Palermo. This is now corrected in the affiliation list.

The authors apologize for these errors and state that they do not change the scientific conclusions of the article in any way. The original article has been updated.

